# Assessment of Chemical, Physicochemical, and Lipid Stability Properties of Gelled Emulsions Elaborated with Different Oils Chia (*Salvia hispanica* L.) or Hemp (*Cannabis sativa* L.) and Pseudocereals

**DOI:** 10.3390/foods10071463

**Published:** 2021-06-24

**Authors:** Carmen Botella-Martínez, José Ángel Pérez-Álvarez, Estrella Sayas-Barberá, Juana Fernández-López, Manuel Viuda-Martos

**Affiliations:** IPOA Research Group, Agro-Food Technology Department, Centro de Investigación e Innovación Agroalimentaria y Agroambiental (CIAGRO-UMH), Miguel Hernández University, Orihuela, 03312 Alicante, Spain; c.botella@umh.es (C.B.-M.); ja.perez@umh.es (J.Á.P.-Á.); estrella.sayas@umh.es (E.S.-B.); j.fernandez@umh.es (J.F.-L.)

**Keywords:** gelled emulsion, hemp oil, chia oil, pseudorecereals, fat replacer

## Abstract

Gelled emulsion (GE) systems are one of the novel proposals for the reformulation of meat products with healthier profiles. The aims of this research were (i) to develop gelled emulsions using pseudocereal flours (amaranth, buckwheat, teff, and quinoa) and vegetable oils (chia oil, hemp oil, and their combination), (ii) to determine their chemical composition, physicochemical properties, and lipid stability, and (iii) to evaluate their stability during frozen storage. The results showed that GEs are technologically viable except for the sample elaborated with teff flour and a mix of oils. The lipid oxidation was not greater than 2.5 mg malonaldehyde/kg of sample for any of the samples analyzed. The physicochemical properties analyzed showed both the pH and color values of the GEs within the range of values obtained for the fat of animal origin. The texture properties were affected by the type of oil added; in general, the firmness and the work of shear increased with the addition of the mixture of both oils. The samples elaborated with buckwheat and chia oil and quinoa and chia oil had the highest emulsion stability values, which remained among the highest after freezing. The results showed that gelled emulsions, based on chia oil, hemp, and their mixture with pseudocereal flours, are a viable alternative as a possible substitute of saturated fat in the development of novel foods.

## 1. Introduction

In developed countries, and even in developing countries, there is a rising concern on the part of health authorities on how diet can cause numerous diseases [1]. Therefore, several epidemiological studies have exposed that the consumption of diets with high quantities of fat (>40% of energy from fat) and with a high content in saturated fatty acids induces many health-related disorders [2]. Thus, one of the most effective behaviors to reduce the risk to develop several diseases is restraining the consumption of saturated fats. In this sense, meat and meat products are one of the principal dietary sources of saturated fats. These fats, which comprised between 30 and 50% of the product, are rich in saturated fatty acids and cholesterol, and they are considered a promoting factor in the development of several diseases including coronary heart disease, metabolic syndrome, obesity and overweight, inflammation, oxidative stress, etc. [2,3].

As a result of these undesirable health effects of excessive fat consumption, the meat industry has had to adapt to these consumers’ requirements developing low-fat meat products with healthier lipid profiles. To achieve this objective, besides the reduction of fat content, numerous strategies have been tried including (i) the direct addition of vegetable oils with healthier lipid profiles [4,5], (ii) the incorporation of vegetable oils with healthier profile encapsulated in several matrices [6,7], (iii) the use of oleogels [8,9], and (iv) the use of gelled emulsions [10,11]. A gelled emulsion is a colloidal material in which oil-in-water emulsion (O/W) coexists within a gel network. Its formation consists of two stages; in the first, the O/W emulsion is elaborated, and in the second stage, the gelled emulsion is properly obtained with the formation in the aqueous phase of a drop structure of the emulsion inside of the cross-linked structure of biopolymers [12]. To elaborate these O/W emulsions, several vegetable or marine oils, as well as mixes of them with a healthier fatty acid profile have been utilized, including chia oil, linseed oil, tiger nut oil, and algal oil, among others [10,11,12,13,14]. Nevertheless, it should be borne in mind that reducing or replacing the fat content in a meat product is not an easy task. Animal fat is a basic ingredient in the processing of meat products due to its technological (to improve emulsion stability, the impact on rheological and structural capacities, and the adjustment of the drying process in dry-cured meat products, among others) and sensory properties (positive effects on hardness, juiciness, color, tenderness, palatability, and so on) [15,16]. Additionally, the addition of vegetables or marine oils with a healthier fatty acid profile may cause an acceleration of lipid oxidation reactions, which can lead to a decrease in the product shelf life as well as a deterioration of their sensorial and nutritional properties [17].

As mentioned above, several vegetable oils can be used to elaborate gelled emulsion. Chia (*Salvia hispanica* L.) oil is a significant oilseed due to its nutritional composition, consisting of up to 65% α-linolenic acid and 20% linoleic acid in the unsaturated fatty acid fraction [18]. Since 2014, it can be marketed in the European Union. On the contrary, hemp (*Cannabis sativa* L.) oil is not widespread on the market, although it is also characterized by an interesting fatty acid composition with a high content of polyunsaturated fatty acids. Thus, in this composition, it is possible to find a high content, up to 75%, of polyunsaturated fatty acids and the unique ratio of 3:1 between omega-6 and omega-3. Hemp oil highly contains linoleic acid and α-linolenic acid in the range of 50–60% and 20–25%, respectively [19]. In addition, there are high amounts of chlorophyll in the oil due to the harvesting of high amounts of immature seeds [20].

With the objective to stabilize the O/W emulsion formed, several ingredients (mainly starchy ingredients) have been used. Pseudocereal flours (from quinoa, amaranth, buckwheat, teff, etc.) seem to be excellent candidates for this application [21,22]. They contain high-quality proteins, abundant amounts of starch with unique characteristics, large quantities of micronutrients such as minerals, vitamins, and bioactive compounds, and they are gluten-free, which makes them suitable for people suffering from various gluten intolerances. Their main component, starch, has many interesting features such as very small granules ready to form cross-link structures, which made them useful for stabilizing emulsions [23]. For these reasons, interest in pseudocereals has increased immensely since the turn of the century, and research efforts have been intensified to include them in our diet. Therefore, the objective of this work was (i) to develop gelled emulsions using pseudocereal flours (amaranth, buckwheat, teff, and quinoa) and vegetable oils (chia oil, hemp oil, and their combination at 50%), (ii) to determine their chemical composition, physico-chemical properties, and lipid stability, and (iii) to evaluate their stability during frozen storage.

## 2. Materials and Methods

### 2.1. Plant Material

Chia oil (CH) was obtained from Herbolarios Navarro, (Alicante, Spain), while hemp oil (H) was purchased from Laboratorios Almond, S.L. (Librilla, Spain). Amaranth flour (A) was obtained from Tentorium Energy S.L. (Tarragona, Spain); buckwheat flour (BW) and white quinoa flour (WQ) were purchased from Biogran S.L. (Madrid, Spain), and whole teff flour (T) was obtained from El granero integral, S.L. (Madrid, Spain). The gelling agent was gellan gum (an extracellular polysaccharide excreted by microorganism *Pseudomonas elodea*). It is a water-soluble linear structure with a repeating unit of tetrasaccharide) and instant gel (gelatin of animal origin (pork) with 180 bloom), which was obtained from Sosa Ingredients S.L. (Barcelona, Spain).

### 2.2. Lipid Profile of Vegetable Oils

The identification of fatty acids was carried out according to the method 969.33 [24]. For that, fatty acids of all samples were transmethylated producing fatty acid methyl esters (FAME). The FAMEs were analyzed on HP 6890 chromatography equipment with a flame ionizer detector and a Suprewax-280 capillary column (30 m, 0.25 μm of film, 0.25 mm internal diameter; Tecknokroma Barcelona, Spain). The injector and detector temperatures were 250 and 270 °C respectively. The temperature program was as follows: the initial temperature was 60 °C, and this was maintained for 1 min after the injection; subsequently, it was raised at a rate of 10 °C/min until reaching 170 °C and was kept at this temperature for 2 min. After these 2 min, it was raised at a speed of 3 °C/min until reaching 230 °C, and it was kept at this temperature for 10 min, and finally, it was raised at a speed of 2 °C/min until reaching 260 °C and maintained for 1 min at this temperature. The carrier gas was helium with an internal column pressure of 11 psi. The injector volume was 0.2 μL in splitless. The response factors were calculated using fatty acid standards, and their identification was made by comparison with the retention times of these FAME standards (Supelco 37 component FAME Mix, Bellefonte, PA, USA). With the data obtained from the chromatograms, the following parameters were calculated: total saturated fatty acids (SFAs), total unsaturated fatty acids (UFAs), total monounsaturated fatty acids (MUFAs), total polyunsaturated fatty acids (PUFAs), the ratio between saturated and unsaturated fatty acids (SFAs/UFAs), and the ratio between omega-3 and omega-6 fatty acids (ω-3/ω-6). All analyses were carried out in triplicate (three independent batches), and the results were expressed as g fatty acid/100 g oil. 

### 2.3. Gelled Emulsions Preparation

Gelled emulsion (GE) preparation essentially involves producing a protein-stabilized emulsion using emulsifying agents and incorporating a gelling agent such as a hydrocolloid or other ingredients with the gelling capacity to convert the emulsion into a GE. Twelve different types of oil-in-water (O/W) GE samples were formulated, as shown in Table 1. Eight GE samples were made combining each of the flours with each of the oils: amaranth flour with chia oil or hemp oil (ACH and AH, respectively); buckwheat flour with chia oil or hemp oil (BWCH and BWH, respectively); whole teff flour with chia oil or hemp oil (TCH and TH, respectively) and finally, white quinoa flour with chia oil or hemp oil (WQCH and WQH, respectively). For the other four GE, a blend of chia oil and hemp oil (50:50 *v*/*v*) was made, and each flour was combined with this oil blend. The other four GEs elaborated were amaranth flour with chia and hemp oils blend (AM); buckwheat flour with chia and hemp oils blend (BWM); whole teff flour and with chia and hemp oils blend (TM), and white quinoa flour with chia and hemp oil blends (WQM).

The O/W GE samples were prepared as follows. For each type of GE, first the gelling agent “instant gel” was mixed in a homogenizer (Thermomix 31, Vorwerk-España M.S.L., S.C., Spain) with water for 2 min at 60 °C at high speed. Then, the flour was added and mixed for 1 min at medium speed. In the next step, the temperature was turned down to 37 °C and gellan gum was added and mixed for 2.5 min at 250 rpm. In the last step, the mixture was mixed with the gradual addition of the appropriate amount of oils or their blends for 5 min, at 37 °C and 1100 rpm. The elaborated GEs were placed in metal containers and stored at 4 °C for 20 h until use. The whole process was replicated three times (three independent batches).

### 2.4. Gelled Emulsion Analysis

#### 2.4.1. Proximate Composition

Protein, fat, ash, and moisture content were determined on GE samples using the appropriate methodology from the Association of Official Analytical Chemist [24]. Protein content was determined by the Kjeldahl method with a factor of nitrogen of 6.25. The Soxhlet method was used for fat content determination, with petroleum ether as the extractant. Ash content was determined by incinerating the samples at 525 °C, while moisture was determined by heating the samples in an oven until constant weight.

#### 2.4.2. Physicochemical Properties

The pH of GE samples was measured using a Crison combination electrode connected to a pH-meter Crison model 510, (Barcelona, Spain). These measures were made directly into the emulsion.

The texture of each sample was evaluated using a TA-XT2i texturometer (Stable Micro Systems, Surrey, England). The “Measure Force in Compression” Test was selected, and the accessory TTC spreadability rig (HDP/SR, Stable Micro Systems) was used. It is composed of a 90° male cone probe and five cone-shaped product holders that were precisely matched females. Both cones were 25 mm apart, and the sample was placed into the female cone and pressed down to eliminate air pockets. Any excess sample was scraped off with a knife to leave a flat test area. GE samples were stabilized at 5 °C for 30 min before testing and were forced to flow out at 45° with a test speed of 3 mm/s. During compression, the force increases up until the point of maximum penetration depth. This force value was taken as the “firmness (N)” at this specified depth. The “work of shear (N.s)” represents the total amount of force required to perform the shearing process [25,26]. 

### 2.5. Stability of Gelled Emulsion during Frozen Storage

Since GE samples should be kept frozen until their application to avoid quick oxidation (high unsaturated fat content), it has been decided to assess the influence of freezing time on several properties related to their stability (resistance of emulsion characteristics to changes over time) such as emulsion stability (retention of fluids in the system at maximum levels), color, and lipid oxidation. For that, each emulsion was placed into Petri dishes that were covered, sealed with parafilm, and frozen at −23 °C in an air freezer W7 8210 0X (Whirlpool, MI, USA) for 15 days. After that, samples were thawed in refrigeration conditions (1 h), and color parameters, emulsion stability, and lipid oxidation were assessed as described below.

#### 2.5.1. Emulsion Stability

The emulsion stability was determined following the procedure from [27] with slight modifications. Samples were introduced into centrifuge tubes of 15 mL and centrifuged at 3000 rpm for 1 min. Then, they were heated in a water bath for 30 min at 70 °C and cooled at room temperature; after that, they were centrifuged again at 3000 rpm for 3 min. The samples were left standing upside down to release the separated fat and water onto filter paper. The results are expressed in g of total fluid expelled/100 g of sample and were calculated using the following expression:

(1) $$\%\mathrm{TEF}=\frac{\mathrm{Weight}\;\mathrm{of}\;\mathrm{tube}\;\mathrm{with}\;\mathrm{sample}-\mathrm{Weight}\;\mathrm{of}\;\mathrm{tube}\;\mathrm{with}\;\mathrm{pellet}}{\mathrm{Weight}\;\mathrm{of}\;\mathrm{sample}}\times 100,$$

#### 2.5.2. Instrumental Color Analysis

The instrumental color parameters of GE samples were measured in the CIEL*a*b* color space using a Minolta CM-700 (Minolta Camera Co., Osaka, Japan), with illuminant D65, SCI mode, and an observer angle of 10°. Low reflectance glass (Minolta CR-A51/1829-752) was placed between the samples and the equipment. The CIEL*a*b* coordinates determined were L* (lightness), a* (red/green), and b* (yellow/blue). The magnitudes h°* (hue) and C* (chrome) were calculated with Equations (2) and (3), respectively.

(2) $$\;\;C^{*}=\sqrt{a^{*2\;}+b^{*2}},$$

(3) $$\;\;h{{}^{\circ}}^{*}=arctg\left(\frac{b^{*}}{a^{*}}\right),$$

#### 2.5.3. Oxidative Stability

The oxidative stability of emulsions was evaluated by measuring changes in thiobarbituric acid reactive substances (TBARs). TBARs determination for each sample was performed in triplicate by the method described by Rosmini et al. [28]. TBARs values were calculated from a malonaldehyde (MA) standard curve and were expressed as mg MA/kg sample. 

### 2.6. Statistical Assay

The whole process was replicated three times (three independent batches). Each replication was performed on a different production day, and each batch was analyzed in triplicate. Means and standard deviations of data obtained from the analysis of GE samples are shown in corresponding tables. A one-way ANOVA test and the Tukey-b post hoc test were used to determine significant differences in both the different types of GE samples and the different times of frozen storage. SPSS version 24.0 was used (SPSS Inc., Chicago, IL, USA) for the evaluations at a significance level of *p* < 0.05.

## 3. Results and Discussion

### 3.1. Fatty Acid Profile of Oils Used for Gelled Emulsions Preparation

The fatty acid profile of chia oil, hemp oil, and their blend is shown in Table 2. Linoleic acid was the most abundant (*p* < 0.05) fatty acid in hemp oil (54.44%) followed by α-linolenic acid (19.95%) and oleic acid (8.23%). On the other hand, chia oil showed mainly (*p* < 0.05) α-linolenic acid (56.61%) followed by linoleic acid (17.43%) and oleic acid (15.05%). However, in the blend of these oils, the predominant fatty acids were α-linolenic acid (38.04%) and linoleic acid (36.11%), followed by oleic acid (11.60%).

Polyunsaturated fatty acids (PUFAs) were the most abundant fatty acids in all samples. Hemp oil showed the highest PUFAs content—hardly 6% more than chia oil, which showed the lowest content. In contrast, the ratio between saturated fatty acids (SFAs) and unsaturated fatty acids (UFAs) was the same, without significant differences between samples (*p* > 0.05). Due to the particular composition of these oils, the ω-3/ω-6 was higher for chia oil (3.24) than for hemp oil (0.43). Thus, the blend of both oils showed an intermediate ratio of 1.09. There is an agreement regarding the need to lower the ω-6/ω-3 ratio, and according to some authors, the ideal ratio may be 1:1 or 2:1. However, it can be stated that an adequate intake of both fatty acids, ω-6 and ω-3, is essential for good health and for reducing the percentage of cardiovascular diseases—although it is not clear whether the ratio between them is of any use [29]. The American Heart Association (AHA) published a review recommending the amount of ω-6 to represent between 5 and 10% of total energy consumed. The AHA indicates that the consumption of ω-6 from vegetable oils, nuts, and seeds is beneficial when forming part of a healthy diet plan in which saturated and trans-fats are replaced by PUFAs [30].

Regarding the fatty acid composition in chia oil, higher amounts of most of the saturated fatty acids compared with those obtained in this work have been reported in studies carried out with chia oil directly extracted from chia seeds [31,32]. These authors also reported lower amounts of stearic acid and similar amounts of behenic acid than the values obtained in this work. In general, regarding unsaturated fatty acids, a greater amount of oleic acid, linoleic acid, and α-linolenic acid was obtained in the present study compared to those reported by these authors [31,32,33]. Regarding hemp oil, a similar fatty acid profile has been reported by Abdollahi et al. [19] for oils obtained from four hemp cultivars in the north of Iran. However, the study of Montserrat de la Paz et al. [34] on refined hemp oil showed a higher amount of saturated fatty acids and monounsaturated fatty acids. The fatty acid profile of oils is highly influenced by the raw material (variety, growth conditions, harvest conditions, etc.) and extraction procedure [35]. Despite this, the relationship between omega-3 and omega-6 for both oils was similar to that reported in the scientific literature.

Comparing the lipid profile of these oils with those of the main animal fats used as a fat source in meat products (Table 3), it is easy to verify their healthier composition. Animal fats showed SFA content higher than 25% compared to percentages not higher than 10.3 in these oils, and their PUFA content was lower than 22% compared to more than 74% found in these oils. Given that, it is expected that their use (as GE) for fat replacement in meat products would improve their lipid profile toward healthier one.

### 3.2. Gelled Emulsions 

**Figure 1:**
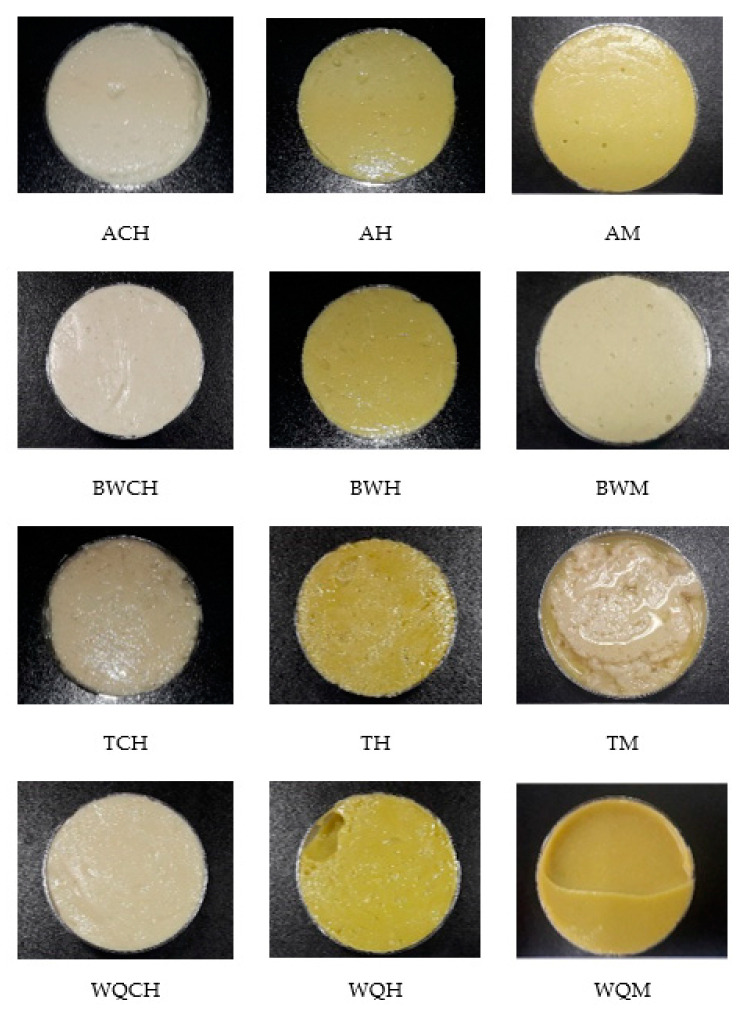
Appearance of the twelve oil-in-water GE samples developed. ACH: amaranth flour with chia oil; AH: amaranth flour with hemp oil; AM: amaranth flour with a blend of both chia and hemp oils; BWCH: buckwheat flour with chia oil; BWH: buckwheat flour with hemp oil; BWM: buckwheat flour with a mix of both oils; TCH: teff flour with chia oil; TH: teff flour with hemp oil; TM: teff flour with a mix of both oils; WQCH: white quinoa flour with chia oil; WQH: white quinoa flour with hemp oil; WQM: white quinoa with a mix of both oils.

Figure 1 shows the twelve GE samples obtained. As can be seen in the figure, the only formula that did not achieve a correct emulsion of its ingredients was TM. It can be clearly seen that the oil was not integrated into the structure of GE. For this reason, TM was no longer subjected to the following analyses and was not considered for further studies.

In the other samples made with teff flour (TCH and TH), a slight oil release can be seen, although both GEs maintained their structure. The samples made with amaranth (AM, ACH, and AH) and buckwheat (BWM, BWCH, and BWH) showed a firmer consistency and showed no noticeable syneresis or oil release. In the same way, very similar GEs were obtained with samples elaborated with white quinoa (WQM, WQH, and WQCH). The structure of each GE and the interactions between its different components are very complicated, and this fact determines their physical properties; any disequilibrium between them seems to be enough to destabilize the systems, among other characteristics [12,27,45]. In this sense, the high molecular weight, as well as branching degree of polysaccharides, plays the role of emulsifying capacity through steric hindrance and charge repulsion [46]; it should be noted that the stability of emulsifiers agents is also affected by many factors such as the heat variability of free proteins and sensitivity to pH [47].

#### 3.2.1. Proximal Composition of Gelled Emulsions 

The proximal composition of GE samples is shown in Table 4. The moisture content of the GEs ranged from 44.73% to 49.91%. For the same flour, moisture content increased (*p* < 0.05) with the addition of the oils blend, except for the amaranth flour that did not present statistically significant differences (*p* > 0.05). 

The highest fat content was 43.86% for the WQCH sample (at the same significance level as WQH, ACH, and BWCH) and the lowest was 35.55% for the TH sample. In general, the highest moisture content and the lowest fat content (*p* < 0.05) were for both oils with teff flour (TCH and TH). Considering that all the emulsions have the same amount of water and oil (47 and 40%, respectively), the differences in water and fat content found seem to be related to the process of the emulsion’s formation. It is possible that the oil or water added to elaborate the emulsion was not perfectly trapped in the gel structure and at the time of sampling, it was not homogeneous. As regards the protein content, GEs elaborated with buckwheat or white quinoa flours as emulsifier agents showed higher values (*p* < 0.05) than EG samples made with amaranth or teff flours. The protein content of GE is determined by the protein content of flour used to make the GE. Regarding the ash content, the GE made with teff flours had the highest (*p* < 0.05) values. No statistical differences (*p* > 0.05) were found between GE samples elaborated with amaranth flour, buckwheat flour, and quinoa flour except for the GE sample elaborated with quinoa flour and blend oils that had the lowest (*p* < 0.05) ash content.

#### 3.2.2. Physicochemical Properties of Gelled Emulsions 

Taking into account that the purpose of these oil-in-water GEs is for them to be used as fat replacers in meat products, is crucial to know their pH value and texture because of the effect on the meat batter formation on the final quality of the meat product. The pH and texture parameters of the GEs are shown in Table 5.

The pH values of all GE samples are in the range of 5.53 to 6.41, which is included within the pH range of the main animal fats (Table 3). The pH of GE samples seems to be related to the type of pseudocereal flour (*p* < 0.05) more than to the type of oil. The lowest pH value (*p* < 0.05) was observed in samples with white quinoa flour (WQM, WQCH, and WQH) and the highest value was observed in samples with amaranth flour (ACH, AH, and AM). The values obtained were lower than those reported by Öztürk-Kerimoğlu et al. [48], who reported that pH values of GEs elaborated with peanut oil and linseed oil as healthier oils and animal protein and inulin as gelling agents were 6.58. Similarly, a study carried out by Verheyen et al. [49] found that the pH value of GEs containing sunflower oil, calcium carbonate, and glucono delta-lactone was 6.34.

Regarding texture parameters, they seem to be mainly affected by the type of oil (*p* < 0.05). For all GE samples, the use of the oils mix (M) significantly increased their firmness (*p* < 0.05) in comparison with the values obtained when only one oil was added. In addition, for the same flour, the GE firmness was higher when chia oil was used than when the oil used was the hemp oil (*p* < 0.05), except for buckwheat flour that did not show differences (*p* > 0.05).

Both texture parameters seem to have similar behavior and have been affected in a similar way for the type of flour and oil. In this way, BWM showed the highest firmness and “work of shear” and WQH showed the lowest (*p* < 0.05). It has been reported that a firmer sample also shows a correspondingly larger area that represents the total amount of force required to perform the shearing process. Both of these values have been shown to rank samples in the same order, but for some samples, many prove to be more suitable than the others [26]. In this case, there are only two samples (AM and WQM) that were not showing the same behavior for firmness and “work of shear”. This could represent that these GE samples need a high peak of force for shearing (high firmness), but once it has been reached, they shear easily and quickly (low “work of shear”).

The rheological behavior of GEs differs widely depending on their composition, structure, droplet interactions, droplet size, etc. [12,50]. Ingredients used for GEs differed in terms of protein content and type, starch content and type, lipid profile, and the presence of other compounds. For example, it has been reported that the proteins in the different pseudocereals used possess suitable emulsifying and gel-forming capabilities [51,52,53]. In this way, protein–protein, protein–oil, and oil–oil interactions driven by hydrogen and covalent bonds, electrostatic, hydrophobic, and electrostatic interactions affect gel strength (protein–protein, protein–oil, and oil–oil) [54]. Finally, oil droplet size also has a considerable effect on the texture properties of emulsion gels [45,55]. In view of that, the formation of a stronger network structure in BWM, as evidenced by the highest firmness and “work of shear”, could be due to the synergic effect of both factors, the specific compounds present in buckwheat flour, and the droplet size of the oils mix, contributing to a stronger gel network.

### 3.3. Stability of Gelled Emulsion during Frozen Storage

#### 3.3.1. Emulsion Stability of Gelled Emulsions 

**Figure 2:**
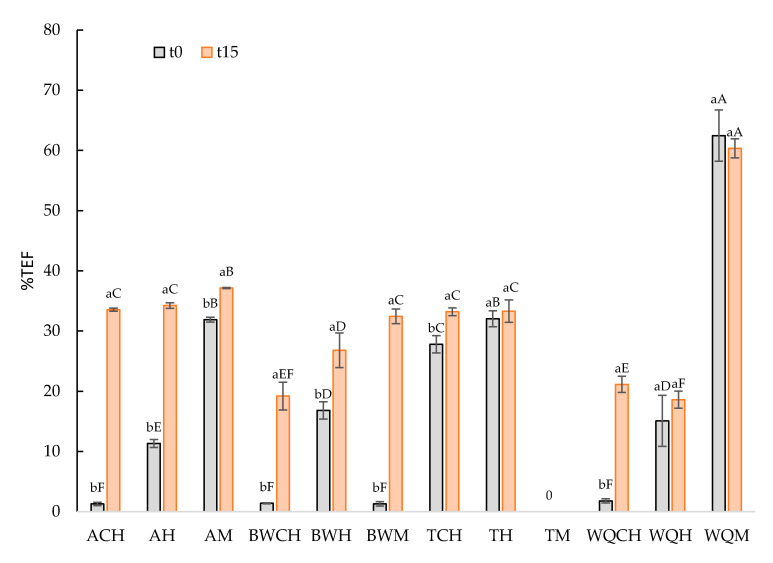
Emulsion stability (%Total Expressible Fluid) of gelled emulsions at day 0 (t_0_) and after 15 days of frozen storage (t_15_). Uppercase letters (A–F) refer to the comparison of the same emulsion stability values and storage time between the different GE samples; lowercase letters (a,b) refer to the comparison of the same emulsion stability values and GE samples between times. ACH: amaranth flour with chia oil; AH: amaranth flour with hemp oil; AM: amaranth flour with a mix of both chia and hemp oils; BWCH: buckwheat flour with chia oil; BWH: buckwheat flour with hemp oil; BWM: buckwheat flour with a mix of both oils; TCH: teff flour with chia oil; TH: teff flour with hemp oil; TM: teff flour with a mix of both oils; WQCH: white quinoa flour with chia oil; WQH: white quinoa flour with hemp oil; WQM: white quinoa with a mix of both oils.

A stable emulsion should retain fluids in the system and also show stable structure at maximum levels: the higher the emulsion stability, the lower the total expressible fluid value. This value is related to several factors such as the water and oil retention capacity, protein–protein interrelations, the amphiphilic properties of proteins, gel structure, cross-linked structure of starch granules, and unsaturated acid fats contents (melting point), among others [56,57]. Some of these factors depend on the flour composition, while others depend on the oil composition and others depend on their interrelation. Other components in pseudocereal matrices can also affect the emulsion properties and stability. Thus, polysaccharides, which are present in a concentration higher than 70 g/100 g in the pseudocereal flours analyzed in this work, can contribute to emulsion stability by crosslinking proteins and adsorbing them at the interface [58]. The presence of lipids in pseudocereals negatively affects the protein emulsifying properties, especially at pH values higher than 6 [59], as occurs in this work. In addition, it is important to notice that at higher protein concentrations in pseudocereal flours (around 12 g/100 g for all flours analyzed in this work), the emulsification properties increases; however, the obtained emulsions are less stable [60]. Figure 2 shows the %TEF of each GE at time 0 (freshly made) and after 15 days of frozen storage. There is not a clear behavior of emulsion stability concerning the type of pseudocereal flour or oil used; it seems that the interrelation between both ingredients would define their effect on the emulsion stability. At time 0, ACH, BWCH, BWM, and WQCH showed the highest (*p* < 0.05) emulsion stability (%TEF < 2.5%), and WQM showed the lowest (TEF > 50%). 

Frozen storage decreased (*p* < 0.05) the emulsion stability in all the GEs except in TH, WQH, and WQM, which kept the same values (*p* > 0.05). ACH and BWM samples showed the highest decrease in emulsion stability due to frozen storage (TEF > 30%). After 15 days of frozen storage, the highest emulsion stability (*p* < 0.05) was found in WQCH, WQH, and BWCH (TEF < 20%) and the lowest was found in WQM (TEF > 50%). The stability of emulsion to freezing and thawing depends on their composition and structure, as well as on the freezing, storage, and defrosting conditions used. The freezing of GEs may crystallize both the oil and water phases, and these phase transitions play an important role in determining the properties of the final products. Depending on the melting point of the fat phase, the fat droplets may crystallize before the water, or vice versa, which can have a major impact on the freeze–thaw stability of a product [61]. In this case, both hemp and chia oils had different melting points, which as mentioned above can affect the stability of the emulsion. Thus, the destabilization of an O/W emulsion using an oil with a high melting point, in which the oil phase crystallizes before the aqueous phase, could be explained due to the coalescence of oil droplets mediated by crosslinking progress during the thawing process, and repeated coalescence eventually leads to the separation of oil and water [62]. The chemical and physical stability of emulsions are influenced by the polymorphism and degree of crystallinity of the lipids, and the phase behavior of water [63]. In turn, these are determined by several factors including emulsifier types, solutes’ composition, and structural, freezing, and processing conditions [64]. 

#### 3.3.2. Lipid Oxidation of GE 

**Figure 3:**
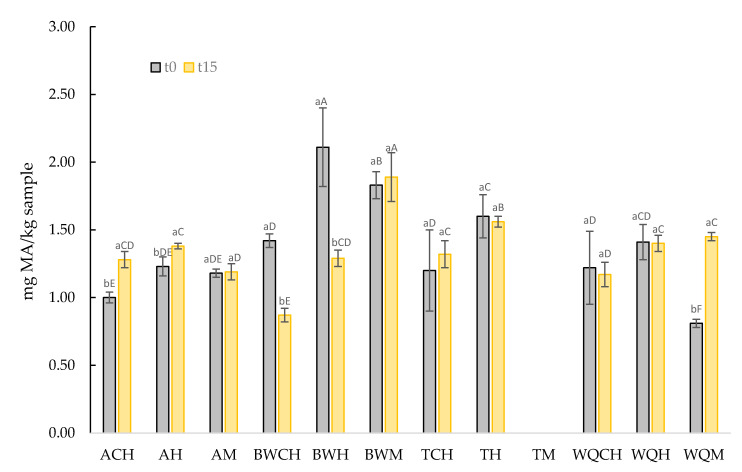
Lipid oxidation (TBARs; mg malonaldehyde/kg sample) of gelled emulsions at day 0 (t0) and after 15 days of frozen storage (t15). Uppercase letters (A–F) refer to the comparison of the same lipid oxidation values and storage time between the different gelled emulsion samples; lowercase letters (a,b) refer to the comparison of the same lipid oxidation values and GE samples between times; results followed by the same lower/uppercase letter are not significantly different according to Tukey’s HSD post hoc test (*p* > 0.05). ACH: amaranth flour with chia oil; AH: amaranth flour with hemp oil; AM: amaranth flour with a mix of both chia and hemp oils; BWCH: buckwheat flour with chia oil; BWH: buckwheat flour with hemp oil; BWM: buckwheat flour with a mix of both oils; TCH: teff flour with chia oil; TH: teff flour with hemp oil; TM: teff flour with a mix of both oils; WQCH: white quinoa flour with chia oil; WQH: white quinoa flour with hemp oil; WQM: white quinoa with a mix of both oils.

In order to monitor the potential oxidation of the new GE developed, which is rich in PUFA, TBARs values at time 0 and after 15 days of frozen storage were measured (Figure 3). It is very important to notice that GE samples had a fat content exceeding 35% with a proportion of PUFA higher than 70%, so a high level of lipid oxidation would be expected. The GE samples showed TBARs values lower than 2.5 mg MA/kg sample both at time 0 and after 15 days of frozen storage. This fact could be explained due to the protein and/or polysaccharide emulsifiers present in pseudocereal flours, which may increase the viscosity of the continuous phase reducing oxygen diffusion and therefore preventing lipid oxidation [65].

In general, GE samples containing amaranth flour (ACH, AH, and AM) or quinoa flour (WQCH, WQH, and WQM) showed lower (*p* < 0.05) TBARs values than GE samples containing buckwheat flour (BWCH, BWT, and BWM) or teff flour (TCH and TH). Considering that the predominant fatty acids are unsaturated fatty acids, which are easily oxidized and that any antioxidant compound has been added in GE formulation, the TBARs values do not seem too high. It must be considered that pseudocereal flours have bioactive compounds, mainly polyphenols as well as tocopherols and tocotrienols, with antioxidants properties, which could be protecting against lipid oxidation [52,53,66,67,68].

In this sense, Antoniewska et al. [69] reported that the addition of a buckwheat/amaranth flours blend into muffins reduces the lipid oxidation degree due to the phenolic compounds as well as phytosterols and tocopherols presents in these flours. Similarly, Jimenez et al. [70] informed that baby dehydrated purees formulated with quinoa and amaranth flours had more fat oxidative stability than control samples due to the bioactive content, mainly tocopherols and tocotrienols, of pseudocereal flours. Previously, Rocchetti et al. [71] reported that pseudocereal flours including quinoa, amaranth, teff, and buckwheat had a high antioxidant capacity measured with FRAP and ORAC assays, and this antioxidant capacity was directly correlated with the content of polyphenolic compounds such as flavonoids (i.e., flavonols) and phenolic acids (hydroxycinnamic). There is not a clear pattern to describe the effect of frozen storage on TBARS; in some cases, their value was increased (ACH, AH, and WQM) or not modified (AM, BWM, TCH, TH, WQCH, and WQH) or even reduced (BWCH, and BWH).

#### 3.3.3. Color Properties of GE 

As the purpose of these GE applications is to be used as the replacement of animal fat, their colors must be as close as possible to the color of pork backfat or lard or even poultry fat (traditional fat sources in meat products). The visual appearance of these GEs (Figure 1) could indicate that GE samples have colors into this range but with clear differences between them. 

Knowing that the color is highly influenced by the development of lipid oxidation, it has been considered interesting to assess their color changes during frozen storage. The color parameters of GEs at day 0 and after 15 days of frozen storage are shown in Table 6.

The lightness values of GE samples ranged between 58.78 and 78.07. All these L* values are in the range of L* reported for animal fats (Table 3). It could be said that L* depends on both main ingredients (flour and oil) because there is not a clear pattern of any of them. L* depends on the water and oil free on the ultrastructure of the product surface: the higher the amount of this ingredient on the surface, the higher the L* values [72]. The water and oil-holding capacity attributed to each flour and the special oil composition could be responsible for these L* variations. All GE samples showed lower L* values after frozen storage. The frozen process modifies the ultrastructure; there are water migrations inside the samples, and also, the water and oil-holding capacity could be modified [64]. All these factors could be affecting L* changes.

Redness (a*) values ranged between 0.19 and 1.48 with differences between them (*p* < 0.05), although it must be considered that differences lower than 1 unit have no practical effect upon visual color. GE samples containing amaranth or quinoa flour seem to have the same behavior in relation to the type of oil added: the addition of hemp oil increased a* values (*p* < 0.05) in relation to chia oil. On the contrary, a* values of GE samples containing buckwheat or teff flour decreased (*p* < 0.05) when hemp oil was added. Hemp oil has a high content of chlorophyll, which could be affecting a* values [20]. These changes could be attributed to the subtraction or addition effect upon red color components determined by the type of oil. All GE samples showed redness values into the range reported for animal fats (Table 3). Although frozen storage caused some variations (increase, reduce, or not variation) in a* values compared to the same values at time 0, any of these differences were higher than 1 unit, so this has no practical importance. 

Yellowness (b*) values ranged between 8.78 and 29.73 with significant differences between them (*p* < 0.05). In this case, a clear effect (increasing) of hemp oil on b* values can be observed in reference to chia oil. The yellow components present in hemp oil would seem to be responsible for this b* increase. Several authors have reported a high content in total chlorophylls (up to 57.66 mg/kg) and carotenes (up to 146.80 mg/kg) in hemp oil [73]. This high carotene content could be contributing to yellowness increase. All GEs showed yellowness values into the range of that reported for animal fats (Table 3).

Frozen storage caused a slight modification in the b* values of GE samples. The behavior of C* in GE samples seems to be related to the b* coordinate (b*-dependent) in both cases: before and after frozen storage. 

GE samples containing quinoa flour (WQCH, WQH, and WQM) showed hue values in the range of yellow hue (90.00°–97.50°). GEs containing teff flour (TCH and TH) showed hue values in the range of yellow-orangish (82.50°–89.99°). GE samples containing amaranth or buckwheat flour showed a hue values range dependent on the type of oil: with chia oil, the hue values were in the range of yellow-orangish, whereas with hemp oil (alone or in the mix with chia oil), their hue values were in the range of yellow hue [74]. In all GE samples, frozen storage caused slight modifications of hue values, or they were not modified.

## 4. Conclusions

The use of pseudocereal flours (amaranth, buckwheat, teff, and white quinoa) and vegetable oils (hemp oil, chia oil, and a blend of both) results in a technologically viable option to elaborate gelled emulsions with a healthier lipid profile (>70% PUFA). The only combination that is not suitable for further application is the use of teff flour with the blend of both oils. Several combinations of all these ingredients allow the elaboration of GE with different firmness that will be useful for their application in different types of foods. If the oxidative stability of the GE is taken as a quality criterion, AM (amaranth flour + blend oils) and WQCH (white quinoa flour + chia oil) samples are the most suitable for the substitution of fat in the development of new foods low in fat or with a healthier lipid profile. On the other hand, TH (teff flour + hemp oil), and WQH (white quinoa flour + hemp oil) show better behavior (emulsion stability) under the frozen and thawing process, which made them suitable for frozen foods. In any case, more studies are needed to improve the stability of the emulsion. Possible alternatives to improve this stability could be to (i) increase the concentration of the emulsifying agent (pseudocereal flours) and reduce the water content, (ii) increase the concentration of the gelling agents and reduce the water content, or (iii) increase the concentration of the emulsifying agent and the gelling agents and reduce the water content. To sum up, the use of these gelled emulsions in foods development brings a new strategy to produce healthy foods. 

## Figures and Tables

**Table 1:** Formulation of oil-in-water gelled emulsion (GE) samples.

Samples	Water	Instant Gel	Gellan Gum	Amaranth Flour	Buckwheat Flour	Teff Flour	Quinoa Flour	Chia Oil	Hemp Oil
ACH	47	1.5	1.5	10	-	-	-	40	-
AH	47	1.5	1.5	10	-	-	-	-	40
AM	47	1.5	1.5	10	-	-	-	20	20
BWCH	47	1.5	1.5	-	10	-	-	40	-
BWH	47	1.5	1.5	-	10	-	-	-	40
BWM	47	1.5	1.5	-	10	-	-	20	20
TCH	47	1.5	1.5	-	-	10	-	40	-
TH	47	1.5	1.5	-	-	10	-	-	40
TM	47	1.5	1.5	-	-	10	-	20	20
WQCH	47	1.5	1.5	-	-	-	10	40	-
WQH	47	1.5	1.5	-	-	-	10	-	40
WQM	47	1.5	1.5	-	-	-	10	20	20

Values expressed as g/100 g. ACH: amaranth flour with chia oil; AH: amaranth flour with hemp oil; AM: amaranth flour with a mix of both chia and hemp oils; BWCH: buckwheat flour with chia oil; BWH: buckwheat flour with hemp oil; BWM: buckwheat flour with a mix of both oils; TCH: teff flour with chia oil; TH: teff flour with hemp oil; TM: teff flour with a mix of both oils; WQCH: white quinoa flour with chia oil; WQH: white quinoa flour with hemp oil; WQM: white quinoa with a mix of both oils.

**Table 2:** Fatty acid profile of hemp oil, chia oil, and their blend, which were used as ingredients for the development of gelled emulsions.

Fatty Acid	Hemp Oil	Chia Oil	Chia/Hemp Oils Mix
C14:0	0.04 ± 0.01 ^a^	0.04 ± 0.01 ^a^	0.04 ± 0.01 ^a^
C16:0	6.17 ± 0.08 ^a^	5.84 ± 0.04 ^b^	6.03 ± 0.01 ^a^
C17:0	0.06 ± 0.01 ^b^	0.05 ± 0.01 ^b^	0.10 ± 0.03 ^a^
C18:0	2.3 ± 0.01 ^c^	3.63 ± 0.09 ^a^	2.98 ± 0.01 ^b^
C20:0	0.94 ± 0.03 ^a^	0.19 ± 0.02 ^c^	0.57 ± 0.02 ^b^
C22:0	0.41 ± 0.01 ^a^	0.1 ± 0.01 ^c^	0.27 ± 0.03 ^b^
C24:0	0.32 ± 0.01 ^a^	0.15 ± 0.01 ^b^	0.14 ± 0.01 ^b^
C16:1 cis	0.1 ± 0.01 ^c^	0.05 ± 0.01 ^b^	0.08 ± 0.01 ^a^
C16:1 trans	0.02 ± 0.01 ^a^	0.01 ± 0.01 ^a^	0.02 ± 0.01 ^a^
C17:1	0.03 ± 0.01 ^a^	0.03 ± 0.01 ^a^	0.04 ± 0.01 ^a^
C18:1 cis	8.23 ± 0.01 ^c^	15.05 ± 0.07 ^a^	11.60 ± 0.04 ^b^
C18:1 trans	0.56 ± 0.01 ^b^	0.62 ± 0.02 ^ab^	0.68 ± 0.03 ^a^
C18:2 (ω-6)	54.44 ± 0.01 ^a^	17.43 ± 0.09 ^c^	36.11 ± 0.14 ^b^
C18:2 (ω-3)	4.26 ± 0.01 ^a^	0.01 ± 0.00 ^c^	2.16 ± 0.01 ^b^
C18:3 (ω-3)	19.95 ± 0.01 ^c^	56.61 ± 0.12 ^a^	38.04 ± 0.06 ^b^
C18:3 (ω-6)	1.62 ± 0.01 ^a^	0.02 ± 0.01 ^c^	0.81 ± 0.03 ^b^
C20:1	0.45 ± 0.01 ^a^	0.13 ± 0.02 ^c^	0.29 ± 0.02 ^b^
C20:2	0.09 ± 0.01 ^a^	0.05 ± 0.01 ^ab^	0.08 ± 0.02 ^a^
C20:3 (ω-11)	0.02 ± 0.01	ND	ND
SFA	10.24 ± 0.08 ^a^	10.00 ± 0.03 ^a^	10.12 ± 0.04 ^a^
UFA	89.77 ± 0.06 ^a^	90.01 ± 0.02 ^a^	89.83 ± 0.09 ^a^
MUFA	9.39 ± 0.02 ^c^	15.89 ± 0.09 ^a^	12.70 ± 0.03 ^b^
PUFA	80.38 ± 0.07 ^a^	74.12 ± 0.08 ^c^	77.20 ± 0.05 ^b^
SFA/UFA	0.11 ± 0.01 ^a^	0.11 ± 0.01 ^a^	0.11 ± 0.01 ^a^
ω-3/ω-6 ratio	0.43 ± 0.03 ^c^	3.24 ± 0.01 ^a^	1.09 ± 0.02 ^b^

Results are expressed as g/100 g. ND: not detected. SFA: saturated fatty acids; UFA: unsaturated fatty acids; MUFA: monounsaturated fatty acids; PUFA: polyunsaturated fatty acids. Results followed by the same lowercase letter are not significantly different according to Tukey’s HSD post hoc test (*p* > 0.05).

**Table 3:** Fatty acid profile, color parameters, and pH of the main animal fats used in meat products.

Parameters	Beef Tallow ^(1)^	Pork Back Fat ^(2)^	Poultry Skin ^(3)^
Lipid profile (% of total lipids)			
C14:0	1–1.5	1–1.5	-
C16:0	24–28	24–28	20–24
C16:1	2–3	2–3	5–9
C18:0	20–24	13–14	4–6
C18:1 (ω-9)	40–43	43–47	33–44
C18:2 (ω-6)	2–4	8–11	18–20
C18:3 (ω-3)	<1	<1	1–2
SFA	46–55	38–43.5	25–31.5
MUFA	42–46	45–50	38–53
PUFA	2–4	8–11	19–22
SFA/UFA	1.0	0.7	0.4
Color parameters			
L*	71.4	71.9	64.6
a*	1.2	3.3	2.6
b*	24.5	7.8	9.9
pH	5.3	6.3	6.0

^(1)^ Motram et al. [36]; Alm [37]; Daly et al. [38]; ^(2)^ Motram et al. [36]; Ospina-E et al. [39]; Jiménez-Colmenero et al. [40]; Méndez-Cid [41]. ^(3)^ Sheu and Chen [42]; Feddern et al. [43]; Alm [37]; Peña-Saldarriaga et al. [44].

**Table 4:** Chemical composition of GE.

Sample	Moisture	Fats	Proteins	Ash
ACH	45.76 ± 0.30 ^cd^	42.82 ± 0.30 ^ab^	2.50 ± 0.03 ^c^	0.43 ± 0.01 ^c^
AH	46.07 ± 0.14 ^c^	42.56 ± 0.10 ^b^	2.52 ± 0.01 ^c^	0.44 ± 0.01 ^bc^
AM	46.10 ± 0.30 ^c^	42.24 ± 0.13 ^b^	2.52 ± 0.02 ^c^	0.48 ± 0.09 ^bc^
BWCH	45.24 ± 0.36 ^cd^	42.69 ± 0.32 ^ab^	2.61 ± 0.01 ^b^	0.49 ± 0.08 ^bc^
BWH	46.18 ± 0.28 ^c^	41.69 ± 0.54 ^c^	2.63 ± 0.02 ^ab^	0.41 ± 0.01 ^c^
BWM	47.59 ± 0.30 ^b^	40.41 ± 0.37 ^d^	2.61 ± 0.01 ^b^	0.45 ± 0.04 ^bc^
TCH	48.28 ± 1.94 ^ab^	37.89 ± 1.23 ^e^	2.47 ± 0.01 ^c^	0.68 ± 0.06 ^a^
TH	49.91 ± 2.57 ^a^	35.55 ± 1.52 ^f^	2.50 ± 0.02 ^c^	0.73 ± 0.05 ^a^
TM	ND	ND	ND	ND
WQCH	44.73 ± 0.06 ^d^	43.86 ± 2.31 ^a^	2.62 ± 0.06 ^ab^	0.46 ± 0.03 ^bc^
WQH	45.33 ± 0.21 ^cd^	43.16 ± 1.97 ^a^	2.68 ± 0.01 ^a^	0.40 ± 0.03 ^c^
WQM	47.15 ± 0.15 ^b^	41.53 ± 0.30 ^c^	2.69 ± 0.02 ^a^	0.26 ± 0.02 ^d^

Results are expressed as g/100 g. ND: not determined. ACH: amaranth flour with chia oil; AH: amaranth flour with hemp oil; AM: amaranth flour with a mix of both chia and hemp oils; BWCH: buckwheat flour with chia oil; BWH: buckwheat flour with hemp oil; BWM: buckwheat flour with a mix of both oils; TCH: teff flour with chia oil; TH: teff flour with hemp oil; TM: teff flour with a mix of both oils; WQCH: white quinoa flour with chia oil; WQH: white quinoa flour with hemp oil; WQM: white quinoa with a mix of both oils. For each assessment, results followed by the same lowercase letter (^a–f^) are not significantly different according to Tukey’s HSD post hoc test (*p*  > 0.05).

**Table 5:** Physicochemical properties of GEs.

Sample	pH	Work of Shear (N·s)	Firmness (N)
ACH	6.38 ± 0.01 ^a^	5.78 ± 0.63 ^b^	6.64 ± 0.64 ^b^
AH	6.41 ± 0.02 ^a^	4.51 ± 0.08 ^c^	5.26 ± 0.60 ^c^
AM	6.35 ± 0.01 ^a^	5.22 ± 0.20 ^b^	11.69 ± 0.52 ^a^
BWCH	6.03 ± 0.02 ^c^	0.82 ± 0.02 ^f^	0.83 ± 0.02 ^f^
BWH	6.06 ± 0.01 ^c^	0.89 ± 0.12 ^f^	0.94 ± 0.08 ^f^
BWM	6.21 ± 0.01 ^b^	11.49 ± 1.18 ^a^	14.70 ± 2.25 ^a^
TCH	6.14 ± 0.01 ^b^	5.34 ± 0.20 ^b^	6.76 ± 1.94 ^b^
TH	6.16 ± 0.01 ^b^	3.56 ± 0.18 ^d^	4.08 ± 0.16 ^d^
TM	ND	ND	ND
WQCH	5.94 ± 0.01 ^d^	4.15 ± 0.12 ^cd^	3.82 ± 0.14 ^d^
WQH	5.98 ± 0.01 ^d^	2.77 ± 0.05 ^e^	2.71 ± 0.92 ^e^
WQM	5.53 ± 0.02 ^e^	3.82 ± 0.03 ^d^	7.22 ± 0.26 ^b^

ND: not determined. ACH: amaranth flour with chia oil; AH: amaranth flour with hemp oil; AM: amaranth flour with a mix of both chia and hemp oils; BWCH: buckwheat flour with chia oil; BWH: buckwheat flour with hemp oil; BWM: buckwheat flour with a mix of both oils; TCH: teff flour with chia oil; TH: teff flour with hemp oil; TM: teff flour with a mix of both oils; WQCH: white quinoa flour with chia oil; WQH: white quinoa flour with hemp oil; WQM: white quinoa with a mix of both oils. For each parameter, results followed by same lowercase letter (^a–f^) are not significantly different according to Tukey’s HSD post hoc test (*p*  > 0.05).

**Table 6:** Color parameters of GE samples (freshly, t_0_) and after 15 days of frozen storage (t_15_).

Samples	t_0_					t_15_				
	L*	a*	b*	C*	*h*	L*	a*	b*	C*	*h*
ACH	74.58 ± 1.52 ^Ba^	0.33 ± 0.06 ^Ba^	10.77 ± 0.28 ^Fb^	10.77 ± 0.28 ^Fb^	88.22 ± 0.35 ^Da^	70.77 ± 0.60 ^Ab^	0.34 ± 0.08 ^Ca^	11.82 ± 0.30 ^Da^	11.83 ± 0.30 ^Da^	88.35 ± 0.34 ^Ca^
AH	69.45 ± 1.91 ^Ca^	−1.03 ± 0.22 ^Fb^	23.52 ± 0.54 ^BCa^	23.54 ± 0.54 ^BCa^	92.52 ± 0.54 ^Aa^	61.83 ± 2.29 ^CDb^	−0.53 ± 0.28 ^Ga^	23.22 ± 1.62 ^Ba^	23.23 ± 1.62 ^Ba^	91.27 ± 0.65 ^Aa^
AM	64.68 ± 2.86 ^Da^	−1.04 ± 0.08 ^Fb^	25.15 ± 2.69 ^Ba^	25.17 ± 2.69 ^Ba^	92.41 ± 0.46 ^Aa^	61.74 ± 0.79 ^Da^	−0.12 ± 0.27 ^Fa^	25.54 ± 1.31 ^Aa^	25.55 ± 1.31 ^Aa^	90.25 ± 0.59 ^Bb^
BWCH	74.27 ± 1.98 ^Ba^	1.23 ± 0.20 ^Ab^	8.78 ± 0.47 ^Ga^	8.87 ± 0.49 ^Ga^	82.57 ± 0.86 ^Fa^	65.28 ± 2.35 ^Cb^	1.51 ± 0.4 ^Aa^	8.85 ± 0.27 ^Fa^	8.98 ± 0.26 ^Fa^	80.28 ± 1.02 ^Eb^
BWH	64.35 ± 0.52 ^Da^	0.56 ± 0.18 ^Eb^	23.20 ± 0.57 ^BCb^	23.21 ± 0.57 ^BCb^	91.37 ± 0.42 ^Ba^	60.09 ± 0.87 ^DEb^	−0.15 ± 0.24 ^Fa^	24.98 ± 2.31 ^ABa^	24.98 ± 2.31 ^ABa^	90.32 ± 0.50 ^Bb^
BWM	78.07 ± 1.73 ^Aa^	0.45 ± 0.14 ^Db^	17.56 ± 0.53 ^Da^	17.57 ± 0.53 ^Da^	91.48 ± 0.47 ^Ba^	65.65 ± 1.30 ^Cb^	0.05 ± 0.18 ^Ea^	16.95 ± 0.94 ^Cb^	16.95 ± 0.94 ^Cb^	89.81 ± 0.62 ^Cb^
TCH	64.50 ± 1.77 ^Da^	1.23 ± 0.12 ^Aa^	11.75 ± 0.17 ^Ea^	11.81 ± 0.16 ^Ea^	84.03 ± 0.64 ^Ea^	59.56 ± 1.39 ^Eb^	0.81 ± 0.10 ^Bb^	11.67 ± 0.36 ^Da^	11.69 ± 0.37 ^Da^	84.50 ± 0.36 ^Da^
TH	58.78 ± 0.80 ^Fa^	0.19 ± 0.09 ^Ca^	22.25 ± 1.01 ^Cb^	22.25 ± 1.01 ^Ca^	89.50 ± 0.22 ^Ca^	54.63 ± 1.76 ^Fb^	0.10 ± 0.13 ^EDb^	22.98 ± 1.35 ^Ba^	22.98 ± 1.35 ^Ba^	90.25 ± 0.33 ^Ba^
TM	ND	ND	ND	ND	ND	ND	ND	ND	ND	ND
WQCH	72.38 ± 2.09 ^BCa^	0.34 ± 0.08 ^Da^	11.70 ± 0.56 ^Ea^	11.71 ± 0.56 ^Ea^	91.69 ± 0.41 ^Bb^	68.98 ± 0.50 ^Bb^	−0.51 ± 0.07 ^Gb^	10.80 ± 0.29 ^Eb^	10.82 ± 0.29 ^Eb^	92.74 ± 0.43 ^Aa^
WQH	62.00 ± 1.02 ^DEa^	−1.16 ± 0.20 ^Fb^	24.67 ± 0.58 ^Bb^	24.70 ± 0.58 ^Bb^	92.70 ± 0.44 ^Aa^	54.12 ± 2.82 ^Fb^	−0.89 ± 0.28 ^Ha^	26.17 ± 1.40 ^Aa^	26.19 ± 1.39 ^Aa^	91.95 ± 0.62 ^Ab^
WQM	60.68 ± 0.50 ^Ea^	1.48 ± 0.14 ^Hb^	29.73 ± 0.77 ^Aa^	29.77 ± 0.77 ^Aa^	92.85 ± 0.23 ^Aa^	58.89 ± 2.25 ^Eb^	−1.09 ± 0.15 ^Ia^	23.41 ± 1.15 ^Bb^	23.44 ± 1.15 ^Bb^	92.66 ± 0.30 ^Aa^

Uppercase letters (A–I) refer to the comparison of the same color parameter and storage time between the different GE samples; lowercase letters (a,b) refer to the comparison of the same color parameter and GE samples between times; results followed by the same lower/uppercase letter are not significantly different according to Tukey’s HSD post hoc test (*p* > 0.05). ND: not determined. ACH: amaranth flour with chia oil; AH: amaranth flour with hemp oil; AM: amaranth flour with a mix of both chia and hemp oils; BWCH: buckwheat flour with chia oil; BWH: buckwheat flour with hemp oil; BWM: buckwheat flour with a mix of both oils; TCH: teff flour with chia oil; TH: teff flour with hemp oil; TM: teff flour with a mix of both oils; WQCH: white quinoa flour with chia oil; WQH: white quinoa flour with hemp oil; WQM: white quinoa with a mix of both oils.

## Data Availability

Data presented in this study are available on request from the corresponding author.
